# PRO^*^MDD Study Protocol: Effectiveness of Outpatient Treatment Programs for Major Depressive Disorder: Metacognitive Therapy vs. Behavioral Activation a Single-Center Randomized Clinical Trial

**DOI:** 10.3389/fpsyt.2018.00584

**Published:** 2018-11-19

**Authors:** Anja Schaich, Laura Heikaus, Nele Assmann, Sandra Köhne, Kamila Jauch-Chara, Michael Hüppe, Adrian Wells, Ulrich Schweiger, Jan Philipp Klein, Eva Fassbinder

**Affiliations:** ^1^Department of Psychiatry and Psychotherapy, University of Lübeck, Lübeck, Germany; ^2^Department of Anaesthesiology, University of Lübeck, Lübeck, Germany; ^3^Division of Psychology and Mental Health, University of Manchester, Manchester, United Kingdom; ^4^Greater Manchester Mental Health NHS Foundation Trust, Manchester, United Kingdom

**Keywords:** major depressive episode, depressive disorders, psychotherapy, metacognitive therapy, behavioral activation, randomized controlled trial, clinical effectiveness

## Abstract

**Background:** Major depressive Disorder (MDD) is a severe mental disorder associated with considerable disability and high costs. Over the last decades, various psychotherapies for MDD have been developed and researched, among others Behavioral Activation (BA) and Metacognitive Therapy (MCT). MCT and BA target different maintaining factors of MDD and have not been compared to date. The PRO^*^MDD randomized controlled trial will compare MCT and BA in the routine clinical setting of an outpatient clinic.

**Methods and Design:** We aim to recruit 128 MDD patients, who will be randomly assigned to either MCT or BA. In both conditions, patients will receive one individual therapy session and one group therapy session per week for a maximum of 6 months. Assessments will take place at baseline, pre-treatment, mid-treatment, post-treatment as well as at 12, 18, and 30 months after start of treatment as follow-up. The primary outcome is reduction of depression severity assessed with the Hamilton Rating Scale for Depression; secondary outcomes address quality of life, psychosocial functioning and participation as well as comorbidity.

**Discussion:** The PRO^*^MDD study is the first randomized controlled trial to compare the effectiveness of MCT and BA. The outcome of this trial will increase our knowledge on the effectiveness and applicability of both treatment modalities and therefore contribute to the improvement of treatment for depressive patients.

**Ethics and dissemination:** The study has been reviewed and approved on 11 August 2016 by the Ethics Committee of the Lübeck University (reference number: 16–176). The results will be discussed through peer-reviewed publications.

**Trial registration:** German Clinical Trials Register DRKS-ID: DRKS00011536 (retrospectively registered)

## Introduction

Depressive disorders range among the most common and disabling psychiatric disorders worldwide. The Global Burden of Disease Study (WHO) identified depression as one of the main causes for disability, accounting for a major part of the global burden of disease (1, 2). The lifetime prevalence of depression has been estimated at 12% in Germany (3) and 17% in the US (4) and the life expectancy of depressed patients is markedly shortened, with women expected to live 12 years and men 16 years shorter than men and women without depressive disorders (5). Approximately 60% of patients that experienced one depressive episode will develop a recurrent depressive episode and every recurring episode increases the risk of further depressive episodes (6–9). About one third of depressive patients develop chronic depression (DSM-5 “persistent depressive disorder”), with depressive symptoms persisting for 2 years or longer (10, 11). Depressive disorders are often accompanied by suicidality as well as psychiatric and somatic comorbidity (12–15). This indicates a great need for adequate treatment of this patient population.

Over the last decades, psychotherapeutic and psychopharmacological treatment options for patients with depression have improved and new treatment methods have been developed and implemented. However, research has indicated that only one in two patients with depression in Germany receives adequate treatment^1^ (16) and one quarter of patients with severe depressive disorders lack specialist outpatient treatment (17). Treatment of depression, especially inpatient treatment, is associated with high direct medical costs and recurrent depressive episodes are associated with a cost increase of 50% (18). Also, depression reduces the level of functioning and participation in working life, therefore accounting for a substantial loss of productivity (17) and indirect costs (18–20). Thus, the development of outpatient treatment programs for depression is much needed. This research study aims to investigate the effect of outpatient treatment programs on depressive symptoms, psychosocial functioning and quality of life by comparing two innovative and promising Behavioral Activation (BA) (21) and Metacognitive Therapy (MCT) (22) .

According to national guidelines (23), patients with depressive disorders are treated with psychopharmacological (antidepressant medication) or psychotherapeutic methods or a combination of both, depending on the severity of the depressive disorder. The importance and efficacy of psychotherapeutic methods in the treatment of depression has been well-investigated and established in over 200 randomized controlled trials (RCTs) (24–27). The method that has been investigated most is cognitive behavior therapy (CBT) which has proved effective compared to a number of control conditions such as waitlist, psychopharmacotherapy, placebo, supportive contacts, and other psychotherapeutic treatment methods. However, remission- and response rates of existing psychotherapeutic treatments for depression indicate that only half of the patients treated by CBT reach remission of their depressive disorder, and that relapse is frequent (28–30). This calls for improvement and optimization of existing treatments for depression.

A promising strategy for optimizing therapy outcome is the development based on research of treatment components which address central psychological mechanisms maintaining depression. Thus, in this study we compare BA and MCT, two methods which target different processes underlying depression, have few overlapping therapy techniques and teach patients a different set of skills (see methods for more details).

Behavioral Activation (BA) is a well-established evidence-based cognitive behavioral method, which has been revised in the course of the “third wave” of CBT (31). BA addresses the loss of positive reinforcements due to avoidance behavior in depression and uses techniques to help patients increase the rate of value-based behavior to foster access to long-term positive reinforcements. In several meta-analyses, the latest including 26 RCTs, BA proved superior to control conditions (waitlist, treatment as usual, psychopharmacotherapy) and comparable to CBT regarding the reduction of depressive symptoms (32–35). The revised manual of BA, which was developed in the course of the “third wave” of CBT, was investigated by a large-scale RCT including 241 patients which compared BA to antidepressant medication and CBT. All treatment methods proved comparable for patients with mild depressive episodes. For patients with moderate and severe depressive episodes, BA and antidepressant medication proved superior to CBT. However, in the BA condition, attrition rate was lower than in the antidepressant condition (36). At 2 year follow-up, patients that responded to therapy showed comparable relapse rates in all conditions. Discontinuation of medication was associated with a poorer prognosis (37).

Metacognitive Therapy (MCT), developed by Adrian Wells at the University of Manchester, UK is a therapy method based on an empirically tested model of psychological disorders. MCT focusses on dysfunctional metacognitive processes central to the development and maintenance of depression (22). Treatment techniques aim to change dysfunctional metacognitions (thoughts/beliefs about cognitive processes) and to reduce related dysfunctional cognitive processes (rumination, worrying, threat-monitoring, and dysfunctional coping strategies) that are summarized as a “Cognitive-Attentional-Syndrome” (CAS). Several research studies showed high effectiveness of MCT in individual as well as in group settings. In small case studies, MCT was associated with a reduction of depressive symptoms after a small number of sessions (38–41). In a small RCT, MCT proved superior to treatment as usual in the reduction of depressive symptoms (42). An Australian RCT conducted independently of the MCT developers compared MCT to CBT. Both conditions showed a reduction of depressive symptoms with moderate to high effect sizes but failed to show a difference between CBT and MCT, which may be due to the small sample size (*N* = 48) (43). A small Iranian RCT including 33 patients with depression compared MCT to CBT and antidepressant medication. MCT and CBT proved more effective than psychopharmacotherapy in the reduction of depressive symptoms, rumination and anxiety (44). MCT seems to be as effective as CBT while requiring fewer therapy sessions. One recent meta-analysis concluded that MCT might be superior to CBT in the treatment of anxiety and depression (45), but more studies are needed.

To date, there are few RCTs on MCT for depression (42–44), and “real-world” studies in regular outpatient therapy settings on both MCT and BA are scarce. Also, there have been no comparative studies on MCT and BA.

The major objective of this study is to compare the effectiveness of MCT and BA in the treatment of patients with moderate or major depressive disorders in a routine German outpatient setting. As this is the first study that compares MCT and BA, it serves as a pilot trial intended to generate preliminary data for orientation and hypothesis generation. The primary hypothesis is that MCT and BA differ significantly in reducing depressive symptoms (two-sided hypothesis). A two-sided hypothesis was chosen because MCT and BA have never been compared to date and the existing literature does not allow predictions on the directionality of any difference between the two methods. Secondary outcomes address general psychopathology, psychiatric comorbidity, quality of life, psychosocial functioning, and attrition. In addition, we undertake a secondary process evaluation to investigate the moderating and mediating factors in BA and MCT that influence outcome. We will also be conducting a time to event analysis as MCT is often said to be particularly quick in inducing remission.

## Methods and design

### Design

The study is designed as a randomized controlled trial (RCT). Patients will be assigned to one of the two active treatment conditions (MCT or BA).

**Figure 1:**
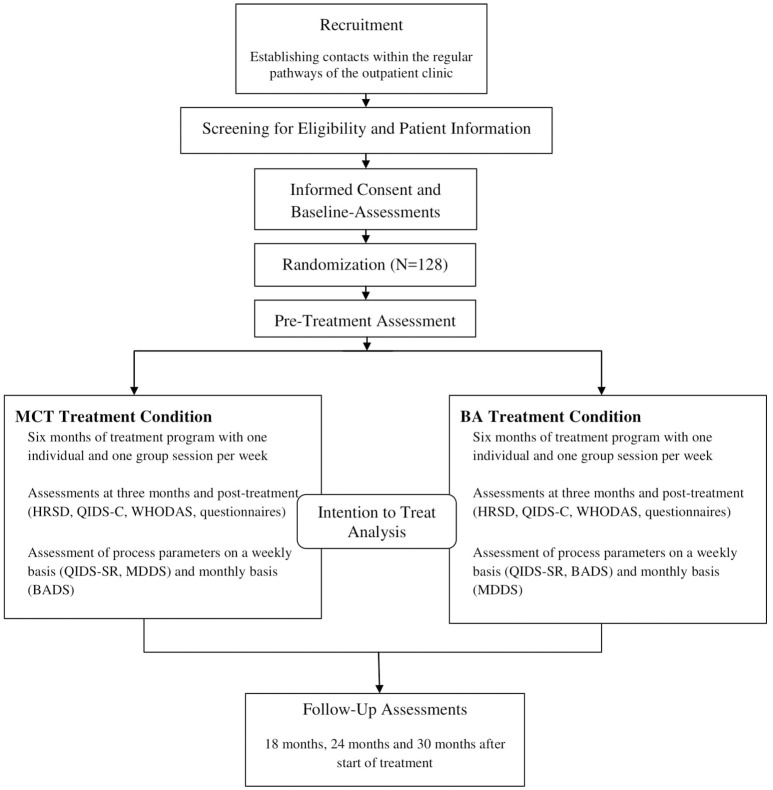
Flow chart of study design. MCT, Metacognitive Therapy; BA, Behavioral Activation.

We aim to investigate the “real world” effectiveness of the two treatments, therefore the PRO^*^MDD study is conducted in the routine clinical setting of an outpatient clinic. As such, we use minimal exclusion criteria, set low barriers for patient participation and guarantee the clinical equipoise of the two treatment programs. Thus, we minimize resistance for participating in a clinical trial with randomization. The PRO^*^MDD trial adheres to the SPIRIT guidelines and methodology (46). For an overview on study design see the flow chart of enrolment, intervention and assessment in Figure 1.

### Setting

Participants will be recruited within the outpatient center of the Department of Psychiatry and Psychotherapy of the Lübeck University Hospital in Germany. The outpatient clinic of the Department of Psychiatry and Psychotherapy treats approximately 510 patients with depressive disorders per year. The department is specialized in treating severely ill patients, often with multiple comorbidities; therefore we included patients with moderate to severe depressive episodes and decided for a maximum treatment duration of 6 months. Potential participants that meet the inclusion criteria are informed about the PRO^*^MDD study during the regular intake procedure verbally and with written information. If they agree to participate and give informed consent, they will be invited for a screening to assess in- and exclusion criteria.

### Eligibility criteria

Patients aged 18 or above will be included if they (1) have a main diagnosis of a depressive episode [as diagnosed by the Structural Clinical Interview for DSM-IV (SCID I)] (2) have a depression severity score of 16 or higher measured by the 24-item Hamilton Rating Scale for Depression (HRSD-24) (3) have read and signed an informed consent form, (4) are able and willing to participate reliably in therapy and assessment procedures. Exclusion criteria are (1) lifetime diagnosis of a psychotic or bipolar disorder, (2) intellectual deficits (IQ < 85), (3) acute suicidality, (4) a main diagnosis other than MDD that requires prioritized treatment and (5) acute substance dependency (according to DSM-5) that requires detoxification treatment. Participation is possible after completion of detoxification treatment and a 2 month period of abstinence.

### Sample size

The primary outcome measure to test the main hypothesis is the severity of depressive symptoms at post-treatment, assessed by the Hamilton Rating Scale for Depression [HRSD, 24-items version (47)]. Confirmatory two-tailed testing of two conditions requires a sample size of *n* = 64 patients in each condition (total *N* = 128 patients). The sample size calculation (48) is based on a power of 80% and a type I error of 5%, as well as an effect size of *d* = 0.5. The estimated between subjects effect size is set according to the one defined by the National Institute of Clinical Excellence (49) for clinical significant difference (*d* ~ 0.5).

### Randomization

After completion of the baseline assessment, participants are randomised equally (1:1) to the two groups (MCT or BA). Randomisation is stratified by gender to avoid biases due to unbalanced gender distribution. Randomization will be conducted by using the program BiaS (11.02) (50). The allocation is concealed from participants and researchers. After randomization, participants are informed about the start date of the treatment. In the first treatment session participants will be informed about which condition and therapist they are assigned to.

### Intervention

Participants will be randomized to either MCT or BA treatment. The structural conditions in both treatment programs are as similar as possible to guarantee clinical equipoise. In both conditions, treatment duration will be a maximum of 6 months with one individual therapy session (50 min) and one group therapy session (90 min) per week. Patients remitted at the mid-treatment assessment (after 3 months of treatment) can end therapy early. After the 12 months follow up assessment, participants see their study therapist for an evaluation session in which the results of the assessment are discussed and in which further treatment will be planned if necessary. Patients will receive 1–2 individual sessions before the start of group treatment to become accustomed to the treatment model and their therapist and to be prepared for the group sessions. The estimated amount of sessions in the program will be 20–24 individual sessions and 24 group sessions. To date, there is little research on the difference between the effectiveness of combined group and individual therapy vs. individual or group therapy only. For several reasons, we decided to offer a combined group and individual therapy program based on our clinical experience: Adding a weekly group therapy session to weekly individual sessions has the advantage of being able to offer psychoeducation and training of specific skills in a group setting, which leaves enough room in the individual sessions to address personal issues and special needs of the patients. Also, as inclusion takes place every 3 months and treatment duration is 6 months, patients that are new to the group can learn from the patients that have already completed 3 months of treatment. As patients treated in the University Hospital display high chronicity, comorbidity and complex symptomatology, we considered group therapy only to be insufficient.

Groups consist of a maximum of 12 patients and will be conducted by two therapists. Group therapy will be offered in a semi-open way and patients can be included in treatment every 3 months. This ensures that patients can start treatment on a regular basis with short waiting times. Eligible patients who cannot participate in group sessions due to professional or educational involvement or family duties are included in the trial and receive individual therapy only. For both conditions, treatment will be conducted following a written protocol (21, 22) by clinicians trained in the treatment method and supervised by local supervisors. The team of BA-therapists are trained and supervised by US. The team of MCT-therapists are trained by AW and supervised by a certified MCT-supervisor that completed the supervisor training by AW. In addition, both teams meet for supervision sessions on a weekly basis. All individual and group sessions will be videotaped and the recordings will be used for supervision and adherence checks (10% of the videos will be rated by blind raters), using adherence scales developed for the two methods (51, 52).

Additional psychiatric management will take place within the treatment program and psychopharmacotherapy will be offered or optimized if necessary in accordance with national guidelines (23): in case of non-response: (1) increase in dosage (if appropriate) (2) augmentation with Lithium (3) augmentation with antipsychotic medication of the second generation or an evidence based combination of antidepressant medication (4) change of antidepressant medication. Psychopharmacotherapy will be entered as a covariate in the final data analysis.

#### Behavioral activation (BA)

Behavioral Activation (BA) is based on Lewinson's reinforcement model of depression (1974), the first behavioral model for the explanation and treatment of depression. Central to this model is the assumption that a low rate of behavior oriented positive reinforcement triggers and maintains depressive symptoms. This is especially maintained by negatively reinforced avoidance behavior (social withdrawal, low activity rate) and a lack of interpersonal skills. Therefore, the main goal of BA is the structured installation of activities and the reduction of behavior that promotes depression. In the course of the “third wave” of cognitive behavior therapy, the original model has been extended. Instead of just scheduling any activities to increase the patient's activity level, BA is now focusing on the development of skills necessary for engaging in personally meaningful, value-based behavior that allows a long-lasting access to positive reinforcers and leads to a fulfilling life. This requires acceptance of aversive experiences and overcoming avoidance strategies that maintain depressive symptoms. The behavioral change is expected to lead to a positive change in thinking and emotions. Important techniques of BA are self-monitoring strategies like weekly activity schedules that help to relate activities to emotional states and help to plan value-based behavior. In the beginning of BA therapy, learning about individual goals and values is essential to identify value-based behavior. Weekly schedules, planning of activities and contingency management are implemented subsequently to facilitate the installation of value-based behavior. Other techniques are training of interpersonal skills and problem-solving skills.

#### Metacognitive therapy (MCT)

Metacognitive Therapy (MCT) was developed by Wells (22) and assumes that dysfunctional over-thinking processes and biased cognitive control are the underlying factors for the development and maintenance of depressive disorders. Central to the concept of MCT are metacognitions (thoughts/beliefs about cognitive processes) and dysfunctional cognitive processes such as rumination, worry and threat-monitoring as well as dysfunctional coping strategies (e.g., avoidance, thought suppression) that are summarized under the term “Cognitive-attentional-Syndrome” (CAS). The CAS is maintained by positive and negative metacognition (e.g., “rumination helps me find a solution to my problems,” “my depressed thinking is a sign I have lost my mind”). Therefore, the aim in MCT is to change positive and negative metacognitions and to reduce the CAS. This is achieved by formulating a case conceptualization and challenging positive and negative metacognitions and the purpose of rumination and other dysfunctional coping strategies. To help the patient regain flexibility of cognitive control, the patient is introduced to the “Attention Training Technique” (ATT). Another important technique of MCT is “Detached Mindfulness” (DM), in which the patient is enabled to experience thoughts and images, as temporary events without the need of further mental and behavioural enagagement (e.g., rumination, suppression, distraction). In this way, DM helps to develop a new attitude toward internal events. DM is illustrated by a range of metaphors and exercises with the goal to teach patients to identify rumination processes and apply DM on thoughts or beliefs that usually trigger rumination or worry processes. The major differences between MCT and BA are summarized in Table 1.

**Table 1:** Major Differences of MCT and BA.

	**MCT**	**BA**
Case formulation and theory	“Top-down” assumption of development and maintenance of depression and treatment: dysfunctional cognitive processes lead to dysfunctional coping strategies such as rumination, worrying, threat monitoring. In treatment, the installation of functional metacognitive processing is expected to promote functional behavior.	“Bottom-up” assumption of development and maintenance of depression and treatment: low activity rate leads to low positive reinforcement which promotes depressed mood. In treatment, the installation of value-based behavior is expected to help patients get access to stable positive reinforcement and by this promote positive change of emotion and cognition.
Trained skills	DM, ATT, rumination postponement, activity scheduling later in treatment if necessary	Self-observation by activity protocols, establishing individual goals and values, value-based activity scheduling, interpersonal skill training, problem-solving skill training
Therapeutic strategies	Case Formulation, use of metaphors, socratic dialogue and exercises to explain and train DM and ATT. Cognitive restructuring of metacognitions.	Validation of depressive behavior and thinking, promoting self-observation-techniques (e.g., week schedules) and behavior analysis, acceptance of aversive emotions, promoting opposite action, activation exercises, detailed planning of activities, behavior-oriented skill-training.
Analysis of problem behavior	Use of the case formulation to demonstrate problematic cognitive processes.	Use weekly schedule to link aversive emotions to problematic behavior.
Structure of individual session	ATT at the start of each session and as homework assignment. Focus on dealing with thoughts and other internal events. Usage of metaphors and exercises to illustrate DM as a different way of reacting on thoughts. Reduction of the CAS by teaching skills for dealing with internal events (DM). Activity scheduling in a later state of treatment.	Checking homework assignment and week schedule at start of session. Use of worksheets to work on individual values, interpersonal and problem-solving skills. Focus on behavior change. Session ends with concrete activity scheduling and homework assignment.
Structure of group session	Introduction activity (e.g., DM exercise) and ATT at the beginning of each session. Focus on reduction of CAS and dealing with thoughts and other internal events.	Homework and goal-related opening and closing round. Focus on activity scheduling and activation, including activating exercise and planning weekly activities in each session.

*MCT, Metacognitive Therapy; BA, Behavioral Activation; DM, Detached Mindfulness; ATT, Attention Training Technique; CAS, Cognitive Attentional Syndrome*.

### Assessments

Assessments are scheduled at baseline, pre-treatment, mid-treatment (3 months after treatment start) and treatment completion (6 months after start). Follow-up assessments will be conducted at 12, 18, and 30 months after treatment start. Assessments include interviews (conducted by blind and independent research assistants and clinicians trained in the interviews) and self-report measures (administered by a computer). Assessment time points are summarized in Table 2.

**Table 2:** Interviews and questionnaires used at each assessment.

	**Baseline**	**Pre-treatment**	**Mid-treatment**	**Post-treatment**	**Follow-up assessments**		
			**3 months**	**6 months**	**12 months**	**18 months**	**30 months**
HRSD	•	•	•	•	•	•	•
QIDS-C16	•	•	•	•	•	•	•
SKID-I	•				•	•	•
SKID-II	•				•	•	•
Structured assessment depression	•			•	•	•	•
WHODAS 2.0	•		•	•	•	•	•
Demographic questionnaire	•			•	•	•	•
Medication questionnaire	•		•	•	•	•	•
CTQ	•					
PHQ-9	•		•	•	•	•	•
BSI	•		•	•	•	•	•
SF-12	•		•	•	•	•	•
MDD-S	•		•	•	•	•	•
MCQ-30	•		•	•	•	•	•
RSQ-D	•		•	•	•	•	•
BADS	•		•	•	•	•	•
DERS	•		•	•	•	•	•
CDQ			•	•		

*HRSD, Hamilton Rating Scale for Depression; SCID I, Structured Clinical Interview for DSM-IV Axis-I Disorders; SCID-II, Structured Clinical Interview for DSM-IV Axis-II Disorders; CTQ, Childhood Trauma Questionnaire; QIDS-C16, Quick Inventory of Depressive Symptomatology; Clinician Rating; PHQ, Patient Health Questionnaire; WHODAS 2.0, World Health Organization Disability Assessment Schedule 2.0; BSI, Brief Symptom Inventory; SF-12, Short Form-12 Health Survey; MDD-S, Major Depressive Disorder Scale; MCQ-30, Metacognitions Questionnaire; RSQ-D, Response Style Questionnaire; BADS, Behavioral Activation of Depression Scale; DERS, Difficulties in Emotion Regulation Scale; CDQ, Care Dependency Questionnaire*.

### Outcome measures

#### Primary outcome measure

*Primary outcome measure* is depressive severity assessed by the 24-item version of the Hamilton Rating Scale for Depression (HRSD-24) (47), a semi-structured interview measuring severity of all symptom domains of depression described by the DSM-IV over the last 7 days. The HRSD has shown good psychometric qualities (47, 53). A cutoff of 16 points has been defined as indicating the presence of a moderate depressive episode and a total score of ≥16 points will be handled as inclusion criterion in this study (54). Remission of depression is defined as an HRSD score of ≤ 8, response as a reduction of 50% in the baseline total score of the HRSD (46, 55) and a score of 15 or less, but of more than 8.

#### Secondary outcome measures

*Secondary outcome measures* are assessed by interview or self-report. They include the following measures.

### Diagnosis of depression and psychiatric comorbidity

Diagnosis of depression and psychiatric comorbidity will be assessed using the German version of the Structural Clinical Interview for DSM-IV (SCID-I and II-Interview) (56, 57). The SCID used in this study will be based on the DSM-IV classification system (58), as the German version of the SCID for DSM-5 is not yet available. Also, a structured assessment of diagnosis and course of depression (59) will be conducted.

### Severity of depression

Severity of depression will be assessed using the HRSD described above as well as the Quick Inventory of Depressive Symptoms (QIDS-C16) (60) and the Patient Health Questionnaire (PHQ-9) (61). All three measures have good psychometric qualities and are sensitive to change (47, 62, 63).

### General psychopathology

General psychopathology will be assessed using the Brief Symptom Inventory (BSI) (64), a short form of the SCL-90-R, which shows good psychometric properties (65).

### General psychosocial level of functioning and participation

General psychosocial level of functioning and participation will be assessed using the WHO Disability Assessment Schedule 2.0 (WHODAS 2.0) (66). The WHODAS 2.0 measures functioning and disability in major life domains (communication and understanding, getting around, self-care, getting along with others, life activities and participation in society). Quality of life will be assessed using the Short Form-12 Health Survey (SF-12) (67).

### Difficulties in emotion regulation skills

Difficulties in emotion regulation skills will be assessed using the Difficulties in Emotion Regulation Scale (DERS) (68). The DERS has high internal consistency, good test-retest reliability and adequate predictive and construct validity (68).

### Traumatic experiences

Traumatic experiences will be assessed only once before start of treatment using the Childhood Trauma Questionnaire (CTQ) (69, 70), which assesses five domains of childhood maltreatment experiences (emotional abuse, physical abuse, sexual abuse, emotional neglect, physical neglect). Both the original and the German version have good psychometric properties (69, 70).

### Care dependency

Care dependency will be assessed using the Care Dependency Questionnaire (CDQ). The CDQ is a reliable and valid questionnaire that measures patient's care dependency. The questionnaire consists of three unidimensional subscales (submissive dependency, need for contact, lack of perceived alternatives) (71).

### Therapists' demographic variables

Therapists' demographic variables will be assessed using the Therapeutic Attitude Scale (ThAt). This questionnaire has been developed by Sandell et al. (ThId) (72) and has been translated into German by Klug et al. (ThAt) (73, 74). In this study, sections A and B of the ThAt, will be used, which assess demographic variables as well as education, supervision and experience.

### Method-specific measures

Method-specific measures will be the Major Depressive Disorder Scale (MDD-S) (22), the Metacognitions Questionnaire (MCQ-30) (67) and the Response Style Questionnaire (RSQ-D) (75) to assess metacognitions, rumination and worry. The Behavioral Activation of Depression Scale (BADS) (76) will be used to assess the degree of behavioral activation.

### Process parameters

The MDD-S, the BADS and the self-rating version of the QIDS (QIDS-SR16) will be used as process parameters. In the MCT condition, the QIDS-SR16 and the MDD-S will be assessed weekly and the BADS monthly. In the BA condition, the QIDS-SR16 and the BADS will be assessed weekly and the MDD-S monthly.

### Psychopharmacological treatment and demographic parameters

Psychopharmacological treatment and demographic parameters will be assessed using a medication questionnaire and a demographic questionnaire.

### Treatment retention

Treatment dropouts are defined as patients who discontinue treatment before remission but agree to be assessed at the planned assessment time points. Study dropouts are patients who drop out of both treatment and assessment. Patients who withdraw their informed consent to participate in the study are study dropouts but may continue treatment as planned. Patients remitted at the mid-treatment assessment (after 3 months of treatment) can end therapy early and are considered “early successes.” Mid-treatment assessment will then be replaced by post-treatment assessment.

### Statistical analyses

Hypothesis will be tested by performing analyis on the whole database including all randomized patients (intention-to-treat analysis). Multiple imputation procedure will be used to replace missing data. We will use linear mixed models (LMM) as they have the advantage of using all available data of each subject. LMM analyses also offer the opportunity to choose an appropriate covariance structure reflecting the potential dependence due to repeated measurements (77). Adjustment for baseline measure will be chosen as this increases statistical power and accounts for regression to the mean (78). All continuous outcomes will be analysed as change from baseline with a random intercept for the participant, time as within-groups factor, study group as well as gender as fixed effect and adjustment for baseline measure. The study hypothesis will be tested on the main effect for group. The estimation of effect size will be based on the imputated dataset and Cohen's d will be calculated for continuous data and numbers needed to treat (NNT) for binary data. The appropriate forms of mixed regression will be chosen for categorical outcome variables, counts and in case of non-normal residuals (poisson, gamma, negative binominal, etc.). To identify predictors or moderators related to treatment outcome, mixed regression methods will be used.

Negative effects will be identified by analyzing and reporting the amount of participants that experienced a clinical significant deterioration [according to Jacobson and Truax (79)].For the time to event analysis, onset of remission will be used as the dependent variable and group assignment as the independent variable. Here, remission will be defined as a score of < 6 on the weekly QIDS-SR16 rating. Patients who do not achieve remission within the 6 months of treatment or dropped out will be censored. No missing data will be substituted for this analysis. The *Kaplan-Meier method* will be used to estimate time to remission (in weeks) and remission rates. The main hypothesis will be tested by comparing the estimated remission rates in both groups using the log-rank test. *Cox proportional hazards regression* will be used to calculate the hazard ratios, to evaluate the influence of covariates for the adjusted analyses and to perform the subgroup analyses.

### Ethical issues

The study protocol is approved by the Ethics Committee of the Lübeck University (reference number: 16–176). Participants will not receive monetary compensation for their participation. All data will be stored securely and pseudonymized. The data collected via computer administered assessments do not include any patient identifiable information. Personal data and video files are stored and secured on servers protected by the university's information technology services. Published material will not contain any patient identifiable information. Protocol amendments or changes will be communicated to relevant parties.

### Risks and benefits

Both study conditions are active psychological treatments with efficacy demonstrated in previous research and without known iatrogenic effects besides side effects of psychotherapy. Participants in this trial will be monitored intensively so that any worsening or suicidal risk can be identified and dealt with immediately. Participants that become suicidal during the course of their participation in this study will receive enhanced therapeutic assistance or will be referred to an inpatient setting within the University Hospital as appropriate. The psychiatric emergency service of our department is available for patients at any time.

### Curent state of the trial

Recruitment, enrollment and treatment of participants has started.

## Discussion

In this article the study design of the PRO^*^MDD trial is described, which is a controlled randomized trial that compares the clinical effectiveness of BA and MCT in the treatment of MDD. The primary hypothesis is that the two treatments significantly differ in reducing depressive symptoms. To the best of our knowledge, the PRO^*^MDD study is the first RCT comparing the effectiveness of BA and MCT in the treatment of MDD.

BA and MCT have different underlying assumptions regarding the development and maintenance of depressive symptoms and use different approaches and treatment techniques. It is therefore hypothesized that there will be differences regarding the main outcome measure (severity of depressive symptoms) as well as psychosocial functioning, quality of life and other secondary outcome measures (rumination and worrying, avoidance behavior, emotion regulation, comorbidity). The two methods will also be examined regarding their effectiveness in different subgroups of MDD patients (e.g., with and without comorbid anxiety disorders or personality disorders, different subtypes of depressive disorders, with or without presence of early trauma). Thus, comparing the two treatment methods can yield important insights to advance our knowledge on psychotherapy for MDD and generate new hypothesis about process parameters leading to change, differential effects, and predictors.

Among the strengths of the PRO^*^MDD study is the common framework that guarantees equipoise of the two study conditions. Also, both treatment conditions work with written manuals that have been used in previous scientific research trials (37, 38, 40–43). In both conditions, treatment is provided by trained therapists under close supervisions and with high allegiance to the specific method. Well-known and established instruments will be used as outcome measures, which allow comparability with other research trials. All outcome measures are assessed by blind, trained, and independent raters. The repeated measurements allow close monitoring of changes over time. The 2 year follow-up period enables the observation of long-term effects of the two treatment methods. Another strength of the PRO^*^MDD study is its implementation in routine clinical care. As minimal exclusion criteria are applied, generalizability and ecological validity are high.

A limitation of the PRO^*^MDD study is that a two-sided primary hypothesis was used instead of a superiority hypothesis of one therapy method or a non-inferiority hypothesis. The reason for choosing a two-sided hypothesis is based on the fact that there is no prior data that justifies a superiority hypothesis of one method above the other, and that for a non-inferiority trial, the power of this study is not expected to be sufficient. As this is the first study to compare BA and MCT for patients with MDD, it intends to generate pilot data for orientation and hypothesis generation for future research to build upon. The sample size was calculated to detect differential treatment effects of a medium effect size. As both treatment methods are known to be effective, between-group differences may be small and therefore not reach statistical significance. Nevertheless, this study will contribute to building a database of comparisons of treatments for MDD which can be used to identify differences in treatment methods using meta-analytic techniques. A further limitation is that the study does not include a control group such as waitlist-control or treatment as usual. Therefore, non-specific factors such as time cannot be controlled for. We decided against an inactive control group for ethical reasons; as most patients treated in this study setting display complex symptomatology, high comorbidity and chronicity, we will try to minimize the time until they can access treatment. Also, a control group with no treatment or treatment as usual might increase resistance of patients and therapists to participate in the trial and this might endanger the representativeness of the study group and the practicability of patient recruitment. Another issue that needs to be discussed is the different treatment durations recommended for the treatment methods. In most MCT studies, treatment duration is a maximum of 10–12 sessions whereas initial studies of BA included up to 24 sessions (36, 80). To guarantee equipoise of the treatment framework, a maximum of 20–24 individual sessions and 24 group sessions is offered to patients in both treatment conditions. In case of early remission, discontinuation of treatment is possible at mid-treatment (after approximately 10–12 sessions). As previous research has shown that a small number of MCT sessions can already lead to substantial improvement in depressive symptoms, it is hypothesized that MCT will lead to a faster symptom reduction than BA.

The PRO^*^MDD study will be conducted in only one center (Lübeck University). This single-center approach has the advantage that adherence to the research protocol as well as organizational and logistic issues are easier to handle than in a multicenter trial. However, it may have implications regarding the generalizability and external validity. As the PRO^*^MDD study will be conducted in an University Hospital setting which treats patients with complex symptomatology and high comorbidity the study population included in this trial will probably show higher symptom severity, chronicity, and comorbidity compared to the general MDD population in Germany.

In conclusion, the PRO^*^MDD study will further our knowledge on the treatment of MDD as both BA and MCT are promising therapeutic methods for this patient group. By investigating both methods in terms of clinical effectiveness but also regarding process parameters, the PRO^*^MDD study will contribute to the development of the best treatment practice for patients with MDD.

## Ethics statement

The study was approved by the ethics committee of Lübeck University. Written informed consent will be obtained from all participants prior to inclusion in the study.

## Data availability statement

Individual patient data will be shared with researchers who provide a methodologically sound proposal. Proposals can be submitted up to 36 months following publication of the main results.

## Author contributions

AS drafting main body of the manuscript; revising manuscript following feedback; involved in organization of logistics, data management, and recruitment of patients. LH involved in organization of logistics, data management and recruitment of patients, provided critical revision of the manuscript. NA involved in organization of logistics, data management and recruitment of patients, provided critical revision of the manuscript. SK involved in organization of logistics, data management, and recruitment of patients, provided critical revision of the manuscript. KJ-C implementation of the two treatment programs in the outpatient clinic, provided critical revision of the manuscript. MH statistical counseling, scientific advisory board, provided critical revision of the manuscript. AW external MCT expert, guards treatment fidelity MCT, provided critical revision of the manuscript. US initial conception and design of the study, development of the study protocol, BA expert, local BA supervision, provided critical revision of the manuscript. JK development of study protocol, expert in treatment of depression, statistical counseling, and recruitment of patients, provided critical revision of the manuscript. EF Coordinating Investigator; initial conception, and design of the study, development of the study protocol. Implementation of the two treatment programs, supervision. All authors read and approved the final manuscript.

### Conflict of interest statement

EF, US, JK, and AW gave trainings and/or published books on MCT and/or BA as well as other psychotherapy models. The remaining authors declare that the research was conducted in the absence of any commercial or financial relationships that could be construed as a potential conflict of interest.

## Footnotes

^1^https://faktencheck-gesundheit.de/de/faktenchecks/depression/ergebnis-ueberblick/.

## Abbreviations

PRO^*^MDD: Programs for Major Depressive Disorder
MDD: Major Depressive Disorder
BA: Behavioral Activation
MCT: Metacognitive Therapy
WHO: World Health Organization
RCT: Randomized Controlled Trial
DSM-5, Diagnostic and Statistical Manual of Mental Disorders: 5th Edition
DSM-IV, Diagnostic and Statistical Manual of Mental Disorders: 4th Edition
CBT: Cognitive behavior therapy
CAS: Cognitive-Attentional-Syndrome
DM: Detached Mindfulness
HRSD: Hamilton Rating Scale for Depression
SCID I: Structured Clinical Interview for DSM-IV Axis-I Disorders
SCID-II: Structured Clinical Interview for DSM-IV Axis-II Disorders
QIDS-C16, Quick Inventory of Depressive Symptoms: clinician-rating
QIDS-SR16, Quick Inventory of Depressive Symptoms: self-rating
BSI: Brief Symptom Inventory
WHODAS 2.0: World Health Organization Disability Assessment Schedule 2.0
SF-12: Short Form-12 Health Survey
MDD-S: Major Depressive Disorder Scale
MCQ-30: Metacognitions Questionnaire
RSQ-D: Response Style Questionnaire
BADS: Behavioral Activation of Depression Scale
DERS: Difficulties in Emotion Regulation Scale
CTQ: Childhood Trauma Questionnaire
NNT: number needed to treat.
